# The Preparation and Characterization of Antioxidant Films Based on Hazelnut Shell-Based Vegetable Carbon Black/Chitosan/Gelatin and the Application on Soybean Oils

**DOI:** 10.3390/foods14101678

**Published:** 2025-05-09

**Authors:** Mengyuan Niu, Jiaxin Wang, Zhaoying Xun, Mengzhuo Liu, He Li, Weiyi Wang, Yuchen Wang, Chao Guo, Hanyu Li, Ning Xu, Huajiang Zhang, Ning Xia

**Affiliations:** 1 College of Food Science, Northeast Agricultural University, Harbin 150030, China; 18846176872@163.com (M.N.); 15134505638@163.com (J.W.); 19845473270@163.com (Z.X.); 19997797983@163.com (M.L.); 13115377712@163.com (H.L.); wangweiyi1999@sina.cn (W.W.); 13009817979@163.com (Y.W.); lihanyu1004@126.com (H.L.); ningxu@neau.edu.cn (N.X.); 2 Heilongjiang Construction Investment Group Co., Ltd., Harbin 150090, China; angelhome2044@sina.com

**Keywords:** hazelnut shell-based vegetable carbon black, active film, mechanical properties, barrier properties, anti-photo-oxidation

## Abstract

In this study, hazelnut shell-based vegetable carbon black (HCB) was synthesized from renewable agricultural waste and incorporated into chitosan (CS) and gelatin (GEL) matrices to fabricate active packaging films. The structure of HCB was characterized, and the structure, physicochemical properties, antibacterial activity, ultraviolet resistance, and functional performance of CS-GEL-HCB films with varying HCB contents (0, 1, 5, and 9 wt% based on GEL) were systematically investigated. The FT-IR results revealed that intermolecular hydrogen bonds were formed between HCB and CS and GEL. The results showed that the tensile strength of CS-GEL film (15.83 ± 0.40~32.06 ± 0.61 MPa), as well as its water vapor and oxygen barrier properties (0.55 ± 0.03~0.15 ± 0.02 g/d·m^2^), and UV-visible light barrier properties were significantly improved (*p* < 0.05) after the addition of HCB, while the water permeability, moisture content, and water solubility of CS-GEL film were effectively reduced (24.84 ± 0.45~20.10 ± 0.45%). More importantly, the CS-GEL-HCB film exhibited enhanced ultraviolet barrier properties, which helped delay the oxidation and deterioration of the oil sample during the accelerated light oxidation test. These results suggest that the CS-GEL-HCB film could serve as an effective food packaging material to improve the oxidation stability of soybean oil in the food industry, showing great potential in maintaining food quality and extending shelf life.

## 1. Introduction

With the increasing public awareness of food safety and environmental sustainability, there has been a growing focus on the development of safe, antibacterial, and antioxidant biodegradable active packaging materials [1,2]. Such degradable green food packaging not only helps preserve food quality but also does not cause environmental damage or pollution [3]. Among the abundant natural biopolymers, chitosan (CS) and gelatin (GEL) have been widely utilized as materials for packaging films, particularly in applications involving meat, fish, vegetables, and fruits, owing to their favorable properties [4,5]. However, both GEL and CS have their own limitations [6,7]. Consequently, the incorporation of CS into GEL can significantly enhance the properties of the resulting packaging material, making it more effective in food preservation and environmental protection practices [8,9,10,11,12]. Therefore, CS-GEL biocomposite films have become a major research focus [13,14,15].

Vegetable carbon black is an edible, food-grade material produced through the carbonization and refinement of vegetable carbon blacks. In the domestic food industry, vegetable carbon black is primarily used as a natural black pigment for coloring confectionery and baked goods. Additionally, its excellent stability and dispersibility make it highly promising for applications in food packaging. It can be incorporated into polymer-based packaging films to enhance their mechanical strength and improve UV-blocking properties [16]. Hazelnut shells, which constitute approximately 50% of the weight of hazelnuts, are considered biomass waste generated during the production and processing of hazelnuts [17,18]. However, hazelnut shells contain a large amount of bioactive substances with potential applications for human health [19,20]. Ozpinar et al. [21] and Haykiri-Acma et al. [22] prepared activated carbon from hazelnut shell waste materials. Hazelnut shells are also a good source for the preparation of vegetable carbon blacks due to their low ash, nitrogen, and sulfur content, coupled with a high fixed carbon content. The resulting hazelnut shell-based vegetable carbon black (HCB) demonstrates excellent photo-oxidation resistance and hydrophobicity, and has the potential to be used as an active packaging additive.

Soybean oil, which is rich in polyunsaturated fatty acids, is prone to oxidation and deterioration under the influence of light and heat in daily life. Many studies have demonstrated that food composite films can delay the oxidation and deterioration of soybean oil [23,24,25]. Ultraviolet radiation significantly accelerates the oxidation and deterioration of soybean oil through the initiation of free radical reactions and photosensitized oxidation. The ultraviolet shielding performance of food composite films plays a critical role in maintaining the stability of oils [25].

In this study, we first prepared vegetable carbon black using hazelnut shell waste and added it to the CS-GEL composite film matrix to develop a new type of composite film. The aim was to investigate the potential effects of the HCB content on the physical and chemical properties, mechanical properties, microstructure, and antibacterial performance of the CS-GEL composite film. Moreover, the influence of this film as a food packaging material on the photo-oxidative stability of soybean oil during storage was studied through a photo-oxidation acceleration test. This research not only realizes the resource utilization of hazelnut shell waste but also provides a new direction for the utilization of vegetable carbon black and the development of food packaging films.

## 2. Materials and Methods

### 2.1. Materials

The hazelnut shells were provided by Tieling Dashen Hazelnut Processing Co., Ltd. (Tieling, China). Soybean oil was obtained from Beidahuang Food Group Co., Ltd. (Harbin, China). CS (degree of deacetylation ≥95%) and P-anisidine (purity: ≥98%) were obtained from Aladdin Biochemical Co., Ltd. (Shanghai, China). GEL (99% Biotech grade), sodium thiosulfate titrant, and starch indicator were obtained from Yuanye Bio-Technology Co., Ltd. (Shanghai, China). All reagents were analytically pure.

### 2.2. The Preparation of Hazelnut Shell-Based Vegetable Carbon Black (HCB)

This experimental method represents the optimal production preparation process obtained after pre-experimental optimization. The hazelnut shells were sequentially washed with deionized water, oven-dried at 105 °C until a constant weight was achieved, then pulverized using a high-speed mechanical mill (DGF-4B, DFT50, Taisite, Tianjin, China), and finally sieved through a 100-mesh standard sieve to obtain uniformly sized particles. The treated hazelnut shell sample was immersed in H_3_PO_4_ solution for activation, and after being drained and dried, it was carbonized at high temperature in a Muffle furnace (SV2–4–10, Bohai, Huanghua, China). After carbonization, the sample was poured into 200–300 mL of 5% HCl solution, boiled at high temperature, and repeatedly rinsed with distilled water and buffer solution (KH_2_PO_4_-Na_2_HPO_4_), repeatedly rinsed until neutral (as indicated by pH meter measurement). This product was then dried at 105 °C until its weight remained unchanged, refined, and pulverized in a superfine grinder (HBM-109, Hanbo, Ruian, China). After sieving through a 200-mesh sieve, the finished product of HCB was obtained following high-temperature sterilization. The morphological structure of the HCB was observed using a field emission scanning electron microscope (SU 8010, Hitachi Company, Tokyo, Japan). The results of the physicochemical property indicators for ensuring the safety of HCB are presented in Table S1 of the Supplementary Materials. The carbon content of the prepared HCB is 97.2%, and the ash content is 2.8%. HCB passed the alkaline soluble chromogenic substance test and the advanced aromatic hydrocarbon test, and the metal element content was also within the standard requirements [26].

### 2.3. The Preparation of CS-GEL-HCB Composite Film

#### 2.3.1. The Preparation of CS Solution and GEL Solution

The optimal concentrations of CS and GEL, as well as the best volume ratio, were obtained based on the results of previous preliminary tests. CS (1.32 g) was dissolved in 60 mL of 2% (*v*/*v*) acetic acid solution, followed by the incorporation of 30 wt% glycerol (relative to CS weight), after which the mixture was subjected to magnetic stirring (800 rpm) at 50 °C for 2 h and subsequently allowed to stand for 1 h under controlled ambient conditions (25 ± 1 °C) until complete bubble dissipation, yielding a uniform CS solution. GEL (0.9 g) was dissolved in 60 mL of deionized water under stirring (500 rpm, 40 °C, 1 h), and then allowed to stand for 1 h until bubble removal, yielding a transparent film-forming solution.

#### 2.3.2. The Preparation of HCB Suspension

Varying HCB contents (0, 1, 5, and 9 wt% based on GEL) were added to 10 mL of deionized water and stirred for 30 min to prepare HCB suspensions with different concentrations. Then, the suspensions were fully homogenized by ultrasound (40 kHz, 30 W) at room temperature for 2 min [16].

#### 2.3.3. The Preparation of Composite Films

The resulting CS solution was slowly added to the GEL solution and stirred continuously for 30 min. Then, different contents of HCB suspension and the CS-GEL film-forming solution were stirred at 45 °C for 1 h. Finally, after degassing, the film-forming solution was uniformly cast onto a rimmed acrylic plate (12 × 12 cm^2^), placed on a horizontal platform using the casting method. After drying at room temperature for 48 h, the films were removed and stored in a desiccator (with a relative humidity of 51% maintained by a Mg(NO_3_)_2_·6H_2_O solution) for preservation. The films with different contents of HCB were named CS-GEL, CS-GEL-1% HCB, CS-GEL-5% HCB, and CS-GEL-9% HCB.

### 2.4. Films’ Structural Characterization

#### 2.4.1. Scanning Electron Microscope (SEM)

Referring to the method of Song et al. [27], the transverse and longitudinal sections of the films were observed at magnifications of ×3 k and ×1 k using a scanning electron microscope (model S-3400 N, Hitachi Ltd., Tokyo, Japan). Before observation, the surface of the sample was treated with a gold coating to facilitate imaging.

#### 2.4.2. Fourier Transform Infrared Spectroscopy (FT-IR)

FT-IR spectra of the films were obtained using a Fourier transform infrared spectrometer (Nicolet iS10, Thermo Fisher Scientific, Waltham, MA, USA) with the Attenuated Total Reflection (ATR) method. A small piece of the composite film (approximately 1 × 1 cm) was cut and carefully adhered flatly to the surface of the ATR crystal. The spectral range was set at 500~4000 cm^−1^, and each sample was scanned 32 times [28].

#### 2.4.3. X-Ray Diffraction Pattern (XRD)

The composite films were cut into 2 × 2 cm squares and fixed with double-sided tape or sample holders to avoid wrinkles. The XRD patterns of the films were examined using an X-ray diffractometer (D8 Advance, Bruker, Karlsruhe, Germany), utilizing Cu Kα radiation within the range of 2θ = 10° to 60° [29].

### 2.5. Optical Properties of the Films

#### 2.5.1. The Appearance and Color of the Films

The films were placed on blank A4 paper. Then, photographs of the films were taken using a digital camera to show their physical appearance. The L, a, and b values of the films were measured using a colorimeter (CR-20, Konica Minolta, Tokyo, Japan). Measurements were conducted individually at the center and at the four corners of the composite film, and the L, a, and b values displayed on the colorimeter were recorded. The total color difference (∆E) and whiteness index (WI) of the film were calculated as follows:

(1) $$\Delta E={\left[{\;\left(\;L^{*}\;-L\;\right)}^{2}+{\left({\;a}^{*}-a\;\right)}^{2}+{\left(b^{*}-b\;\right)}^{2}\right]}^{\frac{1}{2}}$$

(2) $$\mathrm{WI}=100\;-\;{\left[{\left(\;100\;-\;\;L\;\right)}^{2}+{\;a}^{2}+{\;b}^{2}\right]}^{\frac{1}{2}}$$

where L* = 90.22, a* = 1.78, and b* = −11.68.

#### 2.5.2. Transmittance Test

The films (1 × 4 cm) were placed on the side of a cuvette, and a UV-Vis spectrophotometer (UV-2600, Shimadzu, Kyoto, Japan) was used to measure the transmittance of the film samples in the wavelength range of 200–800 nm.

#### 2.5.3. Opacity

The opacity of the composite films was calculated according to the following formula [30]:

(3) $$\mathrm{Opacity}\;\mathrm{Value}\;(mm^{-1})\;=\;\frac{A_{600}}{x}$$

where A_600_ is the absorbance of the film at 600 nm, and x is the average thickness of the film.

### 2.6. Physical Properties of the Films

#### 2.6.1. Thickness of Films

The thickness of the flat, smooth, undamaged, and uniform composite films was randomly measured at five different positions using a digital micrometer (0.001 mm), and the average value was recorded as the thickness of the film.

#### 2.6.2. Mechanical Properties of Composite Films

Generally, elongation at break (EAB) and tensile strength (TS) were used to represent the mechanical properties of the films. Firstly, the composite films were cut into 6 × 1 cm strips, and then the film samples were placed in the TA.XT Plus C texture analyzer, through which the TS and EAB of the films were measured [31]. The formula for calculating TS and EAB was as follows:

(4) $$\mathrm{TS}=\frac{F_{\mathrm{Max}}}{s}$$

(5) $$\mathrm{EAB}=\frac{L\;-{\;L}_{0}}{L_{0}}\;\times \;100\%$$

where F_max_ (N) denotes the maximum tensile force prior to film fracture, s (mm^2^) represents the cross-sectional area of the film, L_0_ (mm) signifies the initial length, and L (mm) corresponds to the length at rupture.

#### 2.6.3. The Moisture Content (MC) and Water Solubility (WS) of Composite Films

Referring to Wu et al. [32], the composite films (2 × 2 cm) were accurately weighed (W_1_) as their initial mass. The films were placed in an oven and dried at 105 °C until the weight no longer changed, then their mass was recorded as (W_2_). After removal, the films were dried again in the oven at 105 °C to a constant weight, weighed (W_3_) again, and the MC and WS of the composite films were calculated according to the following formulas:

(6) $$\mathrm{MC}\;(\%)=\frac{W_{1}-\;\;W_{2}}{W_{1}}\;\times \;100\%$$

(7) $$\mathrm{WS}\;(\%)=\frac{W_{2}-\;W_{3}}{W_{2}}\;\times \;100\%$$

W_1_ represents the initial mass of the film, W_2_ denotes the mass after drying in a drying oven at 105 °C, and W_3_ is the final weight of the film.

#### 2.6.4. WVP of Composite Films

The films (6 × 6 cm) were tightly sealed over weighing bottles containing CaCl_2_ (5 g). The bottles were placed in a desiccator containing saturated NaCl solution and stored at 25 °C for 8 d, and the weight of the weighing bottles was recorded every 24 h. WVP (gm^−1^ h^−1^ Pa^−1^) was calculated according to the following formula:

(8) $$\mathrm{WVP}\;(g\;m^{-1}\;h^{-1}\;Pa^{-1})=\frac{\Delta m\;\times \;d}{A\;\times \;t\;\times \;\Delta P}$$

where Δm represents the mass difference of the weighing bottle (g), A signifies the effective sealed area of the film (m^2^), d denotes the thickness of the composite film (mm), Δt corresponds test time (h), and ΔP stands for the water vapor pressure difference (Pa).

#### 2.6.5. The Oxygen Transmission Rate of Composite Films (OTR)

Referring to Li and Hu et al. [33,34], the mouths of test tubes containing linoleic acid (1 mL) were covered with the composite film and tightly secured with an elastic band. The samples were placed in a storage environment (25 ± 1 °C, 75% relative humidity) for 7 d, and the weight of each test tube was measured every 24 h. Since oxygen can pass through the composite membrane and enter the test tube, it reacts with linoleic acid for oxidation, generating oxidation products that cause the weight to increase. The OTR was determined based on the weight increase of linoleic acid, and the OTR value was calculated according to the following formula:

(9) $$\mathrm{OTR}\;\left(g/d{\cdot m}^{2}\right)=\frac{\Delta m}{S\;\times \;t}$$

where Δm is the weight change of linoleic acid (g), S is the effective area covered by the film (m^2^), and t is the test time of increased time (d).

#### 2.6.6. The Water Contact Angle of Composite Film (WCA)

The WCA of the composite films was determined using an OCA 20 contact angle goniometer (DataPhysics Instruments, Filderstadt, Germany). A total of 5 μL of distilled water was dropped onto the surface of the composite films, and instantaneous images were captured using a high-speed digital camera [35].

### 2.7. The Antimicrobial Activity of Composite Films

According to Li et al. [29], the antibacterial activity of the films against *Staphylococcus aureus* (*S. aureus*) and *Escherichia coli* (*E. coli*) was determined using the agar diffusion method. Firstly, the strains were activated with LB broth medium. Then, an appropriate amount of LB agar medium was poured into the petri dishes and allowed to cool and solidify. Afterward, 200 μL of bacterial solution was evenly distributed on the petri dishes. Subsequently, filter paper discs (6 mm) soaked in the film-forming solution were placed flat on the solidified agar medium and incubated in a 37 °C incubator for 24 h. The antibacterial diameters were observed and measured.

### 2.8. Application in Inhibiting the Oxidation of Soybean Oil

With reference to Ding et al. [16], the photo-oxidation resistance of the composite films during soybean oil storage was evaluated by an accelerated photo-oxidation test. The 50 mL oil sample was divided equally into five open glass vials (20 mL capacity), each containing 10 mL. The four glass vials (including the vials’ mouths and the bodies) were completely wrapped up by the composite films, then fixed with elastic bands, while the remaining vial was left uncovered as a control. The five vials were placed in an airtight opaque box, with a fluorescent lamp (with a power of 20 watts) installed inside, and subjected to irradiation for 30 days. Samples were taken at 10-day intervals to measure the peroxide value (PV), p-anisidine value (AV), and total oxidation value (TOTOX) of the oil.

Among them, the PV of soybean oil was determined by titration. The method for determining AV was as follows: the oil sample was dissolved in isooctane, reacted with an acetic acid solution of p-cresylamine, and the increase in absorbance was measured at a wavelength of 350 nm.

The oxidative rancidity of fats and oils was assessed using TOTOX values, which represent the sum of the peroxide value and p-anisidine value weighted appropriately. The TOTOX value was calculated using the following formula:

(10) TOTOX=2PV+AV

where TOTOX is the total oxidation value of the oil, PV is the peroxidation value of the sample (meq/kg), and AV is the anisidine value of the sample.

### 2.9. Statistical Analysis

All results are presented as the mean ± standard deviation. One-way ANOVA and independent sample *t*-test were conducted using IBM SPSS 26.0 software. Differences were considered statistically significant when *p* < 0.05. Data visualization was performed using Origin 2021 software.

## 3. Results and Discussion

### 3.1. The Characterization of Carbon Black in Hazelnut Shell-Based Vegetables

**Figure 1:**
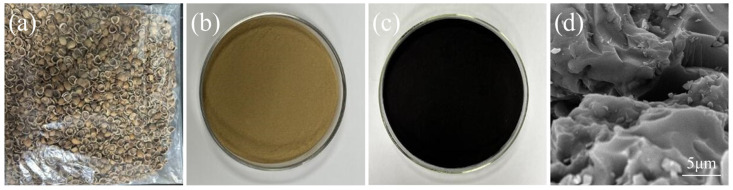
Photos of hazelnut shells after drying (**a**) and hazelnut shell powder (**b**), HCB (**c**), and a SEM image of HCB (**d**).

Figure 1 illustrates the dried hazelnut shells (Figure 1a), the ground hazelnut shell powder (Figure 1b), and the HCB (Figure 1c). The hazelnut shells were washed, dried to a constant weight, and retained their original brown color (Figure 1a). Then, the hazelnut shells were pulverized and sieved through a superfine grinder to obtain the hazelnut shell powder (Figure 1b). The HCB, obtained through a series of processes including phosphoric acid activation, was a black powder that was odorless and tasteless (Figure 1c). SEM results showed that the HCB had micropores and angular prominences on its surface (Figure 1d). The CS and GEL molecular chains may interlock and entangle with these micropores and protruding corners, and it is precisely this interlocking that enhances the barrier properties and tensile strength of the composite film, while reducing elongation at break [16].

### 3.2. The Structural Characterization of Composite Films

#### 3.2.1. Microstructure

**Figure 2:**
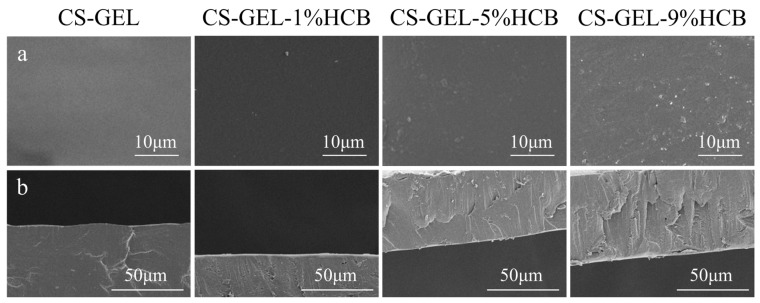
SEM images of the surface (**a**) and the cross-section (**b**) of the films.

Figure 2 shows the microstructure of both the surface and cross-section of the composite films. From the figure, it can be seen that as the amount of HCB added increases, both the planar and cross-sectional profiles of the composite films gradually show a trend of increasing roughness. Wang et al. [36] discovered similar results, where the surface of the composite film gradually became rougher as the amount of watermelon peel extract increased. There is evidence suggesting that as the concentration of carbonaceous particle additives increases, the number of particles will also increase [16]. As the amount of HCB added increased, the cross-sectional profile of the composite films showed a significant increase in roughness. Hanani et al. [37] have also observed that with the increase in the amount of pomegranate peel powder in the film, the structure of the film became discontinuous and the EAB of the film was decreased, which is consistent with the experimental results obtained here.

#### 3.2.2. X-Ray Diffraction Analysis

**Figure 3:**
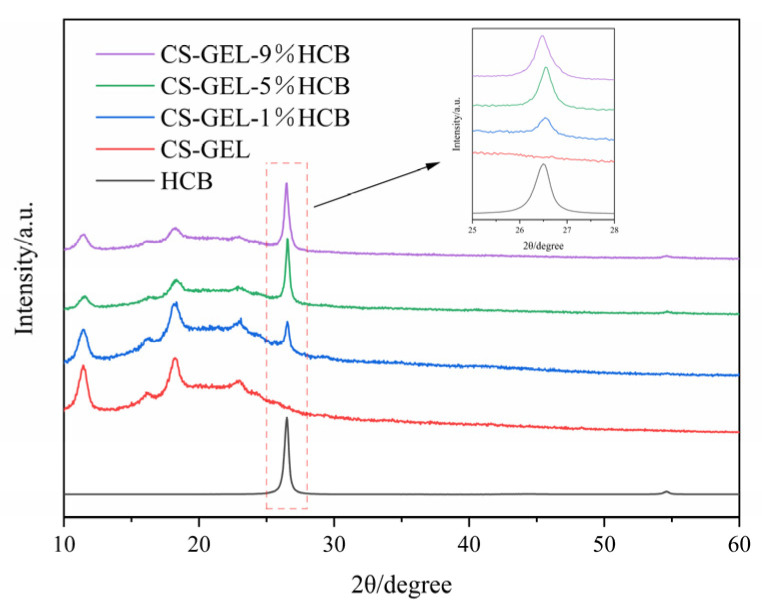
XRD patterns of HCB, CS-GEL, CS-GEL-1%HCB, CS-GEL-5%HCB, and CS-GEL-9%HCB films.

As shown in Figure 3, diffraction peaks of HCB appeared at 26.5° and 43.0°, which corresponded to the (002) and (100) crystal planes of graphite, respectively. This indicates that HCB has a certain degree of graphite-like structure. The crystallinity mainly originated from the graphite microcrystalline structure formed during the high-temperature carbonization of the hazelnut shells. It is the result of the thermal reorganization of biological macromolecules such as cellulose and the reconstruction of the carbon skeleton. Due to the limited degree of graphitization, the peak shape remains broad, indicating that HCB contains amorphous carbon simultaneously. In addition, there were secondary weak peaks near 22.8°and 16.1°, possibly corresponding to the (100) and (004) crystal planes of its layered stacking structure [38]. The main diffraction peak of the CS-GEL composite film appeared at 18.08°, and combined with the background signal of 5~15°, it indicated that CS and GEL formed a partially ordered network structure through hydrogen bonding, resulting in a semi-crystalline material. This was consistent with the diffraction behavior of polysaccharide-protein composite films reported in the literature [39], where the superposition of the amorphous region of gelatin and the (020) crystal plane of chitosan led to peak broadening. After the addition of HCB, the intensity of the diffraction peaks and the crystallinity of the films were enhanced. The crystallinity of CS-GEL gradually increased with the increase of HCB content [40].

#### 3.2.3. FT-IR Spectroscopy Analysis

**Figure 4:**
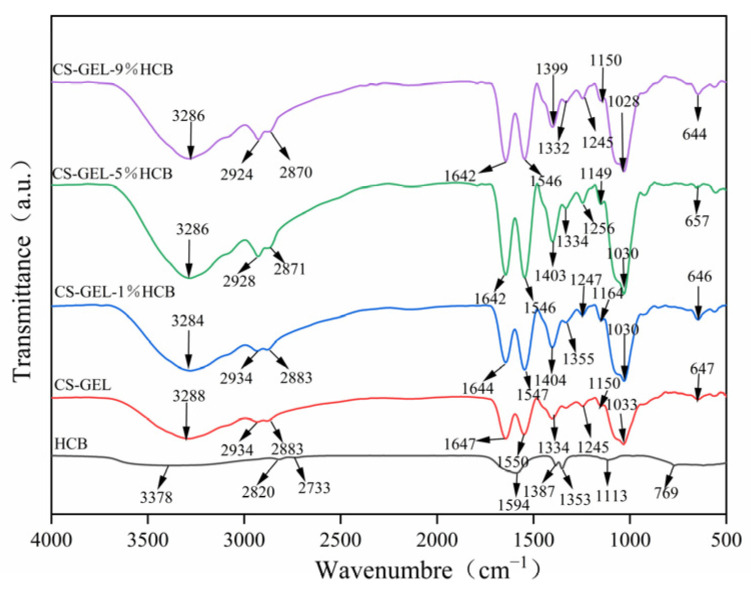
FT-IR spectra of HCB, CS-GEL, CS-GEL-1%HCB, CS-GEL-5%HCB, and CS-GEL-9%HCB films.

Figure 4 shows the FT-IR spectra of HCB and the composite films. The CS-GEL film exhibited characteristic peaks at 3288 cm^−1^, 2934 cm^−1^, and 1642 cm^−1^. Among them, the peaks of 3288 cm^−1^ and 2934 cm^−1^ indicated the formation of hydrogen bonds between CS and GEL. When HCB was added to the composite film, the films exhibited similar main peaks, but with varying intensities. With the increase of HCB content, the peak at 3288 cm^−1^ shifted slightly to a lower wavenumber (3286 cm^−1^), and the peak width increased, suggesting the formation of hydrogen bonds between the hydroxyl group of HCB and the amino/hydroxyl groups of CS-GEL [36]. In addition, slight shifts in the positions of other functional groups (such as the amide I/II bands in the range of 1640–1540 cm^−1^) also supported the existence of hydrogen bonds. Specifically, after adding HCB, the peak at 1647 cm^−1^ (CS-GEL) shifted to 1642 cm^−1^ (CS-GEL-9%HCB), corresponding to the C=O stretching vibration, and the peak at 1550 cm^−1^ (CS-GEL) shifted to 1546 cm^−1^ (CS-GEL-9%HCB), corresponding to the N–H bending vibration. This further illustrated the establishment of intermolecular hydrogen bonds between HCB and the CS-GEL matrix. The FT-IR data analysis of the composite films can also explain the gradual decrease in WVP, WS, and MC with increasing HCB content.

### 3.3. Optical Properties of the Film

**Figure 5:**
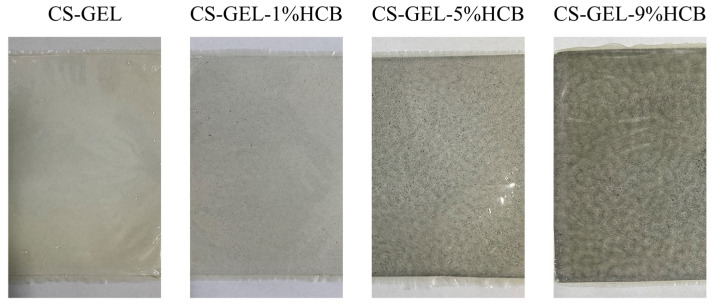
The physical appearances of CS-GEL, CS-GEL-1%HCB, CS-GEL-5%HCB, and CS-GEL-9%HCB films.

The physical appearance of the film is depicted in Figure 5. The CS-GEL composite film appeared nearly colorless and transparent, while the color of the CS-GEL-HCB composite films gradually became darker. With the increase in the amount of HCB added, composite films showed a tendency to turn black, and the opacity gradually increased. This trend may be attributed to the dyeing properties of HCB, which is commonly used as a natural melanin. This observation was consistent with the opacity results of composite films, where a marked increase in opacity was noted with higher HCB content.

Table 1 shows the determination results of the color parameters of the composite films. The values of L, a, b, ∆E, and WI for the composite films with HCB addition were significantly different from those of CS-GEL films (*p* < 0.05). Since HCB was a natural melanin that could stain the film black, the differences in L values among the composite films were analyzed. The L value of the CS-GEL film was the highest, indicating the whitest appearance. With increasing amounts of HCB, the L value of the composite films gradually decreased, indicating that their color became deeper. This could be attributed to the light-absorbing properties of carbon black particles. The ∆E value gradually increased with the addition of HCB content, and the ∆E value of CS-GEL was 3.75 ± 0.13. Whereas for the CS-GEL-9%HCB film, the ∆E value of the film reached 29.09 ± 0.12, demonstrating a significant increase in the contrast with the CS-GEL film and indicating a large color difference between the films. The WI of the CS-GEL film was 86.91 ± 0.02, while that of the CS-GEL-9%HCB film significantly decreased to 62.26 ± 0.17 (*p* < 0.05). The opacity values of the composite films, shown in Table 1, also gradually increased with the addition of HCB. The opacity value of the CS-GEL-9%HCB was 3.85 ± 0.03, significantly higher than that of the CS-GEL film (*p* < 0.05), which was consistent with the observed gradual decrease in the whiteness of the composite films.

**Figure 6:**
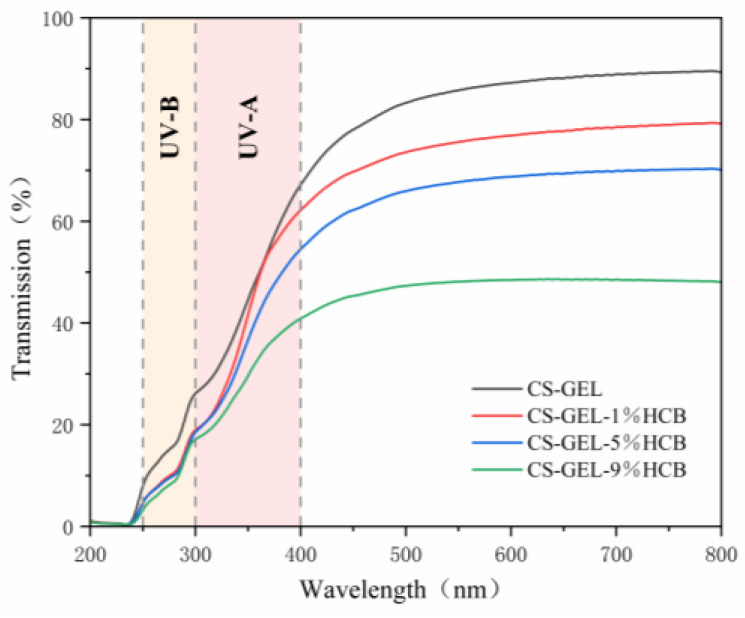
The UV-vis light transmittance of CS-GEL, CS-GEL-1%HCB, CS-GEL-5%HCB, and CS-GEL-9%HCB films.

The light transmittance of the composite films is shown in Figure 6. In the ultraviolet region (200–400 nm), the composite films containing HCB exhibited a notably lower transmittance compared to the CS-GEL film. In addition, the trend of decreasing light transmittance became more pronounced with the increase in HCB addition. In the visible light range (400–800 nm), the transmittance of the composite films with HCB was significantly reduced compared to that of the CS-GEL film. Particularly, the transmittance of the CS-GEL-9%HCB dropped to approximately 40%, whereas the CS-GEL film maintained a transmittance of nearly 90%. This difference may be attributed to the absence of UV-visible absorbing groups in CS and GEL [41]. Meanwhile, the addition of HCB enhanced the anti-ultraviolet and light-blocking abilities of the CS-GEL films. Furthermore, the UV-visible light shielding performance of the composite films was progressively improved with increasing HCB content. This may be due to the ability of HCB to absorb UV and visible light, combined with its surface scattering effects, which together improve the UV-visible light shielding performance of the composite films.

### 3.4. Physical Properties of the Film

#### 3.4.1. Film Thickness and Mechanical Properties

The thickness, TS, EAB, and Opacity value of the composite films are shown in Table 2. It can be seen from the table that the thickness of the CS-GEL composite film was 0.087 ± 0.007 mm. With the increase of HCB addition, the film thickness increased significantly (*p* < 0.05). The main reason for this phenomenon was the increase in the total solid content within the composite film as the amount of HCB added rises [42]. However, when the HCB content exceeded 5%, the film thickness remained relatively unchanged. This suggested that the carbon black particles reached a saturation point, and any excess carbon black particles tended to agglomerate, leading to only a small increase in film thickness [43].

As shown in Table 2, compared with the CS-GEL film, the TS of the composite film with HCB addition was significantly increased. This was consistent with the results shown by the XRD analysis of the composite films. With the increase of HCB content, the crystallinity of the composite films gradually increased, resulting in a more stable structure and a corresponding gradual increase in the TS of the films. With the increasing amount of HCB, this enhancement effect became more pronounced [44].

It can also be seen from the table that compared with the CS-GEL film, the EAB of the composite films gradually decreased with the increase of HCB content. When the addition of HCB exceeded 5%, the EAB of the composite films significantly decreased (*p* < 0.05). This reduction may result from the gradual increase in HCB, which causes more CS and GEL molecules to wrap around the surface of the carbon black particles, limiting their free stretching space. As a result, the film became tighter when subjected to external forces, leading to a decrease in EAB.

#### 3.4.2. The MC, WS, WVP, and OTR of Films

The MC, WS, and WVP of the composite films are shown in Table 3. After the addition of HCB, the MC, WS, and WVP of the CS-GEL film decreased significantly, which may be related to the gradual increase of solid substance content in the composite film. This finding was consistent with the research results of Asif et al. [45], who reported that as the content of fruit peel extract (PPE) increased, the WS and MC of composite films gradually decreased. As the amount of the HCB added increased, the MC of the composite films decreased (*p* < 0.05), likely due to the binding of HCB to CS and GEL, which limited the formation of hydrogen bonds between water molecules and CS-GEL. The HCB contained hydrophobic compounds, contributing to its overall hydrophobicity. Therefore, the decrease in MC reflected the lower water affinity of the CS-GEL-HCB films.

WS reflected the water resistance of the packaging films, as shown in Table 2. With the increase in HCB content, the water solubility of the films decreased significantly (*p* < 0.05). This was consistent with results from other studies, where the increasing content of plant extracts led to a gradual decrease in WS [1,46,47]. The reduced WS of the CS-GEL-HCB film was mainly due to the insolubility of HCB in water. Furthermore, as indicated by the FT-IR results, the intermolecular interactions between HCB and CS-GEL also restricted the hydrophilicity of CS and GEL. As shown in Table 3, the WVP of the composite film showed a trend of gradual decline with the increase of HCB addition. This may be because HCB filled the gap between CS and GEL molecules, making the composite film structure more compact, and thus making it more difficult for water molecules to pass through the composite film matrix. This finding was consistent with the results reported by Wang et al. [48].

**Figure 7:**
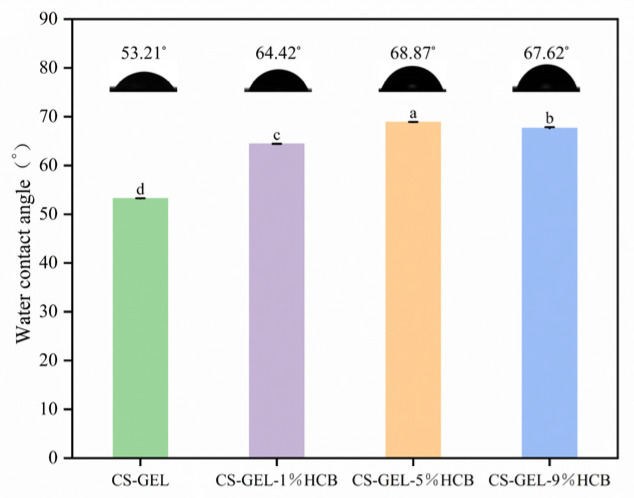
The WCA of CS-GEL, CS-GEL-1%HCB, CS-GEL-5%HCB, and CS-GEL-9%HCB films. Different letters indicate significant differences between the dates (*p* < 0.05).

The OTR of the composite films is also shown in Table 3. The OTR gradually decreased with the increase in HCB content. Compared to the CS-GEL film, the OTR value of the CS-GEL-9%HCB film was significantly lower (*p* < 0.05), which was consistent with the findings of Kadam et al. [49]. This reduction may be attributed to HCB filling the gaps between CS and GEL molecules, thereby making the film structure tighter and enhancing its ability to block the penetration of oxygen molecules into the matrix [50]. The WCA of the composite films is shown in Figure 7. The WCA of each film was less than 90°, indicating that they possessed hydrophilic surface properties. After the addition of HCB to the CS-GEL films, the surface WCA increased significantly, from θ = 53.21° to θ = 68.87°, indicating a significant decrease in surface wettability. This change can be attributed to the complex intermolecular interactions between HCB, CS, and GEL. Such a transition contributed to the stability and barrier properties of the films under humid environments [42]. Similar results were reported by Wang et al. [48], suggesting that the relatively low solubility of HCB in water and the formation of aggregates through intermolecular forces reduced the number of free –OH groups, resulting in an increased WCA of the composite films [51].

### 3.5. The Ability of Film to Inhibit Microbial Activity

The antibacterial capacity of the composite films was usually expressed by the diameter of the inhibition zone. Table 4 shows the diameters of the inhibition zones of the composite films against *E. coli* and *S. aureus*. When the amount of HCB added was less than 5%, the inhibition ability of the composite films against *E. coli* and *S. aureus* significantly increased (*p* < 0.05) with increasing HCB content. On the one hand, this could be attributed to the intrinsic antimicrobial ability of CS, as the cationic amine groups in chitosan bind to the bacterial cell wall, altering its permeability and disrupting normal cellular functions [52]. On the other hand, compared with the CS-GEL film, the antibacterial activity of the composite films supplemented with HCB was higher, which may be attributed to the inherent antibacterial activity of HCB [53]. Thus, the antibacterial effect of the films was significantly enhanced with the increase of HCB content (*p* < 0.05). Previous studies have shown that carbon nanomaterials (CNMs), such as carbon dots, graphene, and activated carbon-based nanocomposites, can serve as antibacterial agents and bio-fillers [54]. However, when the HCB content exceeded 5%, the antibacterial activity of the composite films significantly decreased, which may be due to the agglomeration of excess HCB particles, resulting in a reduction of their effective antibacterial performance.

### 3.6. The Application of Composite Film in Inhibiting the Oxidation of Soybean Oil

#### 3.6.1. The Change of Peroxide Value (PV)

**Figure 8:**
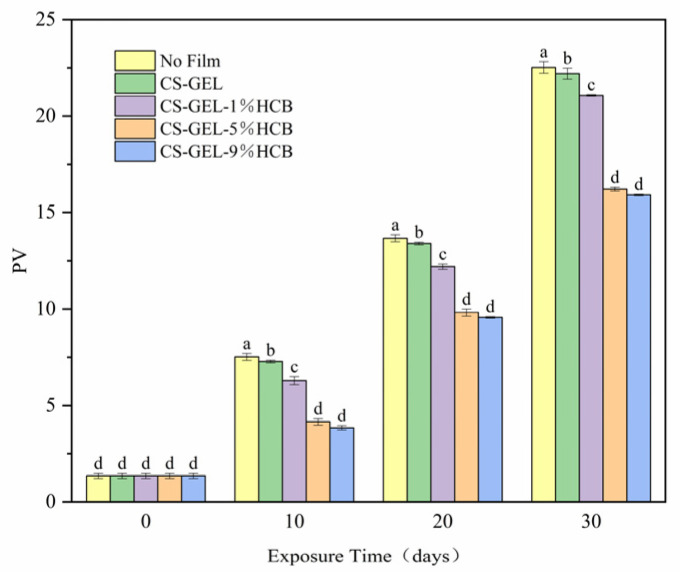
The PV of oil packaged in no film, CS-GEL, CS-GEL-1%HCB, CS-GEL-5%HCB, and CS-GEL-9%HCB film pouches. Different letters indicate a significant difference between the dates (*p* < 0.05).

The PV changes of soybean oil during storage are shown in Figure 8. With the extension of storage time, the peroxide value of soybean oil showed an increasing trend. As shown in Figure 8, compared with the CS-GEL film, the peroxide value of soybean oil coated with the composite film containing HCB exhibited a relatively slower growth trend. Moreover, with increasing HCB content, the ability to delay the growth of the peroxide value in soybean oil was gradually enhanced, which was probably related to the excellent anti-oxidation performance of HCB. These results were consistent with the UV-visible light transmittance data of the composite films. In addition, combined with the OTR results, it can be inferred that HCB filled the gaps between CS and GEL molecules, making the structure of the composite films more compact. Thus, composite films prevented the penetration of oxygen molecules into the film matrix and delayed the oxidation of soybean oil. This finding was consistent with other studies, which have shown that food composite films can effectively delay the oxidation of oils [43].

#### 3.6.2. Change of P-Anisidine Value (AV)

**Figure 9:**
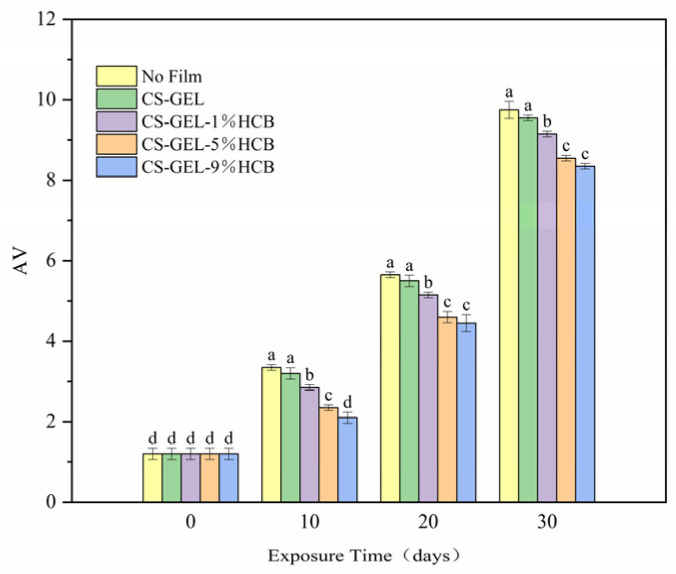
The AV of oil packaged in no film, CS-GEL, CS-GEL-1%HCB, CS-GEL-5%HCB, and CS-GEL-9%HCB film pouches. Different letters indicate a significant difference between the dates (*p* < 0.05).

As shown in Figure 9, the AV of all of the groups of soybean oil exhibited an increasing trend with an extended storage time. It suggested that the primary oxidation products formed during fat and lipid oxidation were further decomposed into secondary oxidation products during storage [55]. Additionally, as the concentration of HCB in the composite films increased, the AV of the corresponding soybean oil gradually decreased, indicating an enhanced ability to delay the oxidation and deterioration. The AV of soybean oil at 0 d was 1.20 ± 0.14. After 30 days, the AV of soybean oil in the control group reached 9.75 ± 0.21, whereas the AV of soybean oil coated with the CS-GEL-9%HCB film was significantly lower at 8.35 ± 0.07 (*p* < 0.05). This significant reduction was mainly attributed to the excellent oxygen barrier properties of the composite films containing HCB, thereby slowing the oxidation and deterioration of soybean oil [56].

#### 3.6.3. Changes in Total Peroxide Value (TOTOX)

**Figure 10:**
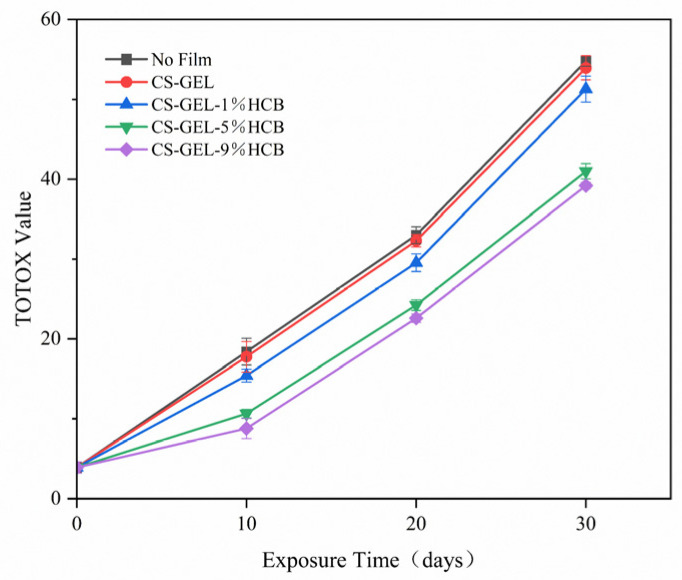
The TOTOX of the oil packaged in no film, CS-GEL, CS-GEL-1%HCB, CS-GEL-5%HCB, and CS-GEL-9%HCB film pouches.

The total oxidation value (TOTOX) combines primary oxidation products, such as hydroperoxides, and secondary oxidation products, including unsaturated aldehydes, to assess the degree of oxidative deterioration of lipids [57]. As shown in Figure 10, the TOTOX of soybean oil increased across all treatment groups following 30 days of accelerated oxidation. After 30 days of storage, the TOTOX values of soybean oil coated with CS-GEL-5%HCB and CS-GEL-9%HCB films were 40.99 ± 0.96 and 40.20 ± 0.39. They were significantly lower than that of the blank control group (*p* < 0.05), indicating that the composite films containing HCB could effectively prolong the storage period of soybean oil. The main reason for this was that the CS-GEL-HCB composite films exhibited excellent photo-oxidation resistance and barrier properties. Previous studies have demonstrated that composite films with superior UV shielding capabilities can effectively delay the oxidation and deterioration of soybean oil [25]. Although the TOTOX value of soybean oil coated with the CS-GEL-9%HCB composite film was the lowest, its effect was not significantly different from that of the CS-GEL-5%HCB composite film. This may be because a higher amount of HCB leads to particle agglomeration, which reduces the ability of HCB to absorb ultraviolet and visible light. Therefore, considering both photo-oxidation resistance and material efficiency, the CS-GEL-5%HCB composite film appeared to be the most suitable packaging material for prolonging the storage of soybean oil.

## 4. Conclusions

After grinding hazelnut shells into powder, HCB was prepared through a series of processes, including phosphoric acid activation, and we subsequently added it to CS-GEL to prepare an active food packaging film. SEM observations showed that the addition of HCB made the surface and cross-section of the CS-GEL film rougher. FT-IR and XRD results confirmed that intermolecular hydrogen bonds were formed between HCB and CS-GEL, and that the TS of the CS-GEL-HCB films was significantly improved by this intermolecular interaction. Due to the hydrophobicity of HCB, the water resistance of the CS-GEL-HCB films was better than that of the CS-GEL films. In addition, the CS-GEL-HCB films exhibited excellent ultraviolet–visible light barrier properties and antibacterial activity, and they could significantly inhibit the oxidation and deterioration of soybean oil during storage. The food composite films demonstrated remarkable anti-photo-oxidation effects on soybean oil during storage.

Notably, the CS-GEL-5%HCB composite film showed great application potential in the field of food packaging, particularly for foods with a high oil content and photosensitivity, such as nuts and fried foods. Overall, as an additive for CS-GEL films, HCB offered the dual advantages of barrier performance and functional activity. This study provided a new strategy for the functional transformation of other waste materials, such as shells or wood.

## Figures and Tables

**Table 1:** The L, a, b, ∆E, and WI of the films.

Films	L	a	b	∆E	WI
CS-GEL	89.91 ± 0.11 ^a^	0.12 ± 0.02 ^d^	−8.33 ± 0.14 ^d^	3.75 ± 0.13 ^d^	86.91 ± 0.02 ^a^
CS-GEL-1%HCB	82.01 ± 0.16 ^b^	0.18 ± 0.02 ^c^	−6.38 ± 0.06 ^c^	9.90 ± 0.10 ^c^	80.91 ± 0.18 ^b^
CS-GEL-5%HCB	74.55 ± 0.27 ^c^	0.26 ± 0.03 ^b^	−5.17 ± 0.12 ^b^	17.04 ± 0.25 ^b^	74.03 ± 0.26 ^c^
CS-GEL-9%HCB	62.41 ± 0.17 ^d^	0.37 ± 0.02 ^a^	−3.28 ± 0.12 ^a^	29.09 ± 0.12 ^a^	62.26 ± 0.17 ^d^

Different letters in the same column indicate significantly different results (*p* < 0.05).

**Table 2:** Thickness, TS, EAB, and Opacity value of films.

Films	Thickness (mm)	TS (MPa)	EAB (%)	Opacity Value (mm^−1^)
CS-GEL	0.087 ± 0.007 ^c^	15.83 ± 0.40 ^d^	49.51 ± 1.03 ^a^	0.67 ± 0.02 ^d^
CS-GEL-1%HCB	0.101 ± 0.003 ^b^	21.26 ± 0.90 ^c^	48.49 ± 0.97 ^a^	0.73 ± 0.01 ^c^
CS-GEL-5%HCB	0.111 ± 0.004 ^a^	32.06 ± 0.61 ^a^	37.11 ± 1.22 ^b^	1.52 ± 0.03 ^b^
CS-GEL-9%HCB	0.117 ± 0.002 ^a^	25.83 ± 0.42 ^b^	32.90 ± 1.20 ^c^	3.85 ± 0.03 ^a^

Different letters in the same column indicate significantly different results (*p* < 0.05).

**Table 3:** The MC, WS, WVP and OTR of films.

Films	MC (%)	WS (%)	WVP (×10^−7^ g m^−1^ h^−1^ Pa^−1^)	OTR (g/d·m^2^)
CS-GEL	16.27 ± 0.44 ^a^	24.84 ± 0.45 ^a^	5.51 ± 0.07 ^a^	0.55 ± 0.03 ^a^
CS-GEL-1%HCB	15.58 ± 0.52 ^a^	23.37 ± 0.67 ^b^	5.14 ± 0.05 ^b^	0.42 ± 0.03 ^b^
CS-GEL-5%HCB	12.27 ± 0.28 ^b^	20.75 ± 0.57 ^c^	3.79 ± 0.16 ^d^	0.23 ± 0.02 ^c^
CS-GEL-9%HCB	11.76 ± 0.22 ^b^	20.10 ± 0.45 ^c^	4.51 ± 0.26 ^c^	0.15 ± 0.02 ^d^

Different letters in the same column indicate significantly different results (*p* < 0.05).

**Table 4:** The antibacterial activity of the films.

Films	Diameter of Inhibition Zone (mm)	
	*Escherichia coli*	*Staphylococcus aureus*
CS-GEL	15.81 ± 0.03 ^c^	18.89 ± 0.06 ^c^
CS-GEL-1%HCB	16.64 ± 0.09 ^b^	19.58 ± 0.06 ^b^
CS-GEL-5%HCB	19.56 ± 0.09 ^a^	22.84 ± 0.07 ^a^
CS-GEL-9%HCB	15.92 ± 0.01 ^c^	18.91 ± 0.07 ^c^

Different letters in the same column indicate significantly different results (*p* < 0.05).

## Data Availability

The original contributions presented in this study are included in the article/Supplementary Materials. Further inquiries can be directed to the corresponding authors.

## Supplementary Materials

The following supporting information can be downloaded at: https://www.mdpi.com/article/10.3390/foods14101678/s1, Table S1: Physical and Chemical Indicators of HCB.
