# Supplementary material for: The Preparation and Characterization of Antioxidant Films Based on Hazelnut Shell-Based Vegetable Carbon Black/Chitosan/Gelatin and the Application on Soybean Oils
Source: Foods. 2025 May 9;14(10):1678. doi: 10.3390/foods14101678 (PMC12110961; doi:10.3390/foods14101678)
Supplement: Supplementary file 1 [file foods-14-01678-s001.zip › foods-3573553-supplementary.pdf]

Table S1. Physical and Chemical Indicators of HCB

Test items	Indicators	Test results
Carbon content (calculated on a dry basis),w/%	≥95	97.2
Dry weight loss, w/%	≤12.0	5.8
Mercury (Hg)/(mg/kg)	≤1	Not detected
Total arsenic (measured as As)/(mg/kg)	≤3	Not detected
Cadmium (Cd)/(mg/kg)	≤1	0.006
Lead (Pb)/(mg/kg)	≤10	0.003
Ash content, w/%	≤4.0	2.8
Advanced aromatic hydrocarbons	Through test	Through test
Alkaline-soluble chromogenic substances	Through test	Through test
